# Measuring Child Socio-Economic Position in Birth Cohort Research: The Development of a Novel Standardized Household Income Indicator

**DOI:** 10.3390/ijerph17051700

**Published:** 2020-03-05

**Authors:** Costanza Pizzi, Matteo Richiardi, Marie-Aline Charles, Barbara Heude, Jean-Louis Lanoe, Sandrine Lioret, Sonia Brescianini, Virgilia Toccaceli, Martine Vrijheid, Franco Merletti, Daniela Zugna, Lorenzo Richiardi

**Affiliations:** 1 Cancer Epidemiology Unit, Department of Medical Sciences, University of Turin, 10126 Torino, Italy; franco.merletti@unito.it (F.M.); daniela.zugna@unito.it (D.Z.); lorenzo.richiardi@unito.it (L.R.); 2 ISER, University of Essex, Colchester CO4 3SQ, UK; matteo.richiardi@essex.ac.uk; 3 Research Team on Early Life Origin (EAROH), Université de Paris, CRESS, INSERM, INRA, F-75004 Paris, France; marie-aline.charles@inserm.fr (M.-A.C.); barbara.heude@inserm.fr (B.H.); jean-louis.lanoe@ined.fr (J.-L.L.); sandrine.lioret@inserm.fr (S.L.); 4 Centre for Behavioural Science and Mental Health, Istituto Superiore di Sanità, 00161 Rome, Italy; sonia.brescianini@iss.it (S.B.); virgilia.toccaceli@iss.it (V.T.); 5 Childhood and Environment Programme, ISGlobal, 08003 Barcelona, Spain; martine.vrijheid@isglobal.org; 6 Department of Experimental and Health Sciences, Universitat Pompeu Fabra (UPF), 08003 Barcelona, Spain; 7 CIBER Epidemiología y Salud Pública (CIBERESP), 28029 Madrid, Spain

**Keywords:** socioeconomic position, income, birth cohorts, children

## Abstract

The assessment of early life socioeconomic position (SEP) is essential to the tackling of social inequalities in health. Although different indicators capture different SEP dimensions, maternal education is often used as the only indicator in birth cohort research, especially in multi-cohort analyses. Household income, as a direct measure of material resources, is one of the most important indicators, but one that is underused because it is difficult to measure through questionnaires. We propose a method to construct a standardized, cross-cohort comparable income indicator, the “Equivalized Household Income Indicator (EHII)”, which measures the equivalized disposable household income, using external data from the pan-European Union Statistics on Income and Living Conditions (EUSILC) surveys, and data from the cohorts. We apply this method to four studies, Piccolipiù and NINFEA from Italy and ELFE and EDEN from France, comparing the distribution of EHII with other SEP-related variables available in the cohorts, and estimating the association between EHII and child body mass index (BMI). We found that basic parental and household characteristics may be used, with a fairly good performance, to predict the household income. We observed a strong correlation between EHII and both the self-reported income, whenever available, and other individual socioeconomic-related variables, and an inverse association with child BMI. EHII could contribute to improving research on social inequalities in health, in particular in the context of European birth cohort collaborative studies.

## 1. Introduction

Socioeconomic inequalities in health have been reported consistently for several outcomes, across the life course and in both low/middle- and high-income countries [1,2,3]. There is evidence that socioeconomic disadvantages in early life not only affect child health but have long-term effects also on adult health independently of adult circumstances [3]. Assessing early life socioeconomic position (SEP) and studying its long-term health influences are therefore essential to tackle population social inequalities in health and to control for confounding when studying outcomes that are strongly socially shaped.

Birth cohort studies follow participants from their fetal life and have the potential to collect information on parental and household social and economic indicators at different time points from the pre-conception period onwards. Such studies are ideally suited to investigate infancy/childhood SEP, which is determined by the SEP of the family of origin. SEP can be measured both at the geographical level, through deprivation indexes, and at an individual level, through different potential indicators; in this paper we will focus on the latter. The individual indicators most commonly used in epidemiological research and potentially available in birth cohorts include education, occupation-based measures and income. Moreover, data on housing characteristics (e.g., size, tenure status), which are measures of material circumstances, are often collected in birth cohorts but rarely employed as main SEP indicators [4].

Maternal education is often used as the only proxy of child SEP [5,6,7,8,9], as it is easy to collect, even retrospectively, it is quite stable over time, it is less affected by childbearing than occupation and income, and it is fairly comparable across different populations and countries, although not across different generations [10]. However, each single indicator (e.g., maternal education, occupation, income) captures different, likely correlated, dimensions of the child SEP [4,8,9,11,12,13]. Using maternal education only, which can be considered as a measure of intellectual resources, might therefore not be the best choice for some research questions (e.g., when studying an outcome strongly influenced by economic/material resources), might be insufficient to control for confounding when SEP is a strong potential confounder of an exposure-outcome relationship, and cannot capture individual changes in SEP over time [10]. Parental occupational based measures, which reflect social standing/prestige and access to economic resources [11], are sometimes used as an alternative or, more rarely, as an additional indicator of child SEP [14,15]. In particular, employment status and occupational class can be collected in birth cohort studies.

The household disposable income is potentially one of the most important single indicators of the child SEP, as it is a direct measure of material resources. However, accurately measuring family income through interviews or self-administered questionnaires might be a difficult task due to several issues. First, income is considered a sensitive matter, and therefore the proportion of (informative) missing values might be higher than for education or occupation; second, it might be difficult for the person who completes the questionnaire to accurately report the income for all family members, increasing the likelihood of measurement errors; finally it is difficult to account for non-salary incomes (e.g., benefits, allowances) and taxes. Moreover, comparing income across populations and studies might be complex, as different studies might collect different types of income (e.g., family disposable income vs. income from work only, net vs, gross etc.) and at different points in time (e.g., before or after birth). This is particularly relevant in the context of international collaborative studies, where it is essential to have harmonized comparable SEP indicators over the different studies.

In this paper we propose, describe and discuss a method for constructing a standardized and comparable cohort-specific household income indicator for child SEP to be used in European birth cohort studies. The indicator uses external data from the pan-European surveys “European Union Statistics on Income and Living Conditions” (EUSILC) [16] and internal data from the cohorts and is constructed using only basic parental and household characteristics, typically available in birth cohort studies, as no actual income data are needed. In this paper we apply this method to four birth cohorts from two different countries, Italy and France.

## 2. Materials and Methods

### 2.1. Data

We used data from the EUSILC survey and from four birth cohort studies. Details on the EUSILC data and on cohort-specific inclusion criteria and study protocols are available in the Supplementary Material.

#### 2.1.1. EUSILC

EUSILC [16] is a survey that collects from 2005 onwards comparable annual microdata at both individual and household level in representative samples of persons aged at least 16 years in 28 European Union States, as well as Iceland, Norway and Switzerland. Individual data can be linked to household data and vice versa. EUSILC has both a cross-sectional and a longitudinal component, but for this study we used the cross-sectional data only. The sample data are based on a nationally representative probability sample of the population residing in private households within the country. The EUSILC survey as data resource for epidemiological research has been described previously [17].

#### 2.1.2. Piccolipiù

Piccolipiù is an Italian multicentre cohort, involving five centers (Turin, Trieste, Firenze, Viareggio, and Roma) that have recruited from 2011 to 2015 about 500 newborns each (1000 in Roma) for a total of approximately 3400 newborns [18].

Data on tenure status, house size (number of rooms), family size, cohabitation status and on parental age, education, occupational status, jobs coded using the ISCO-88 (International Standard Classification of Occupations) classification and country of birth are available. Information on self-reported monthly net total family income in Euros (<1000, 1000–1499, 1500–1999, 2000–2499, 2500–2999, 3000–3999, 4000–4999, 5000–5999, ≥6000; “don’t know”) at the time of completion of the 12-months follow-up questionnaire is also available. Moreover, using the geocoded home addresses at recruitment, the value of a geographical deprivation index has been assigned to each Piccolipiù participant. This is a nationwide deprivation index at municipality and census block level, based on the 2001 Census Italian data [19].

Child weight and height data are collected at each follow-up questionnaire. For this paper we used the measures gathered at the 2- and 4-year follow-up visits, restricting the analyses to those children with body mass index (BMI) measured between 20 and 28 months and 46 and 54 months of age, respectively.

#### 2.1.3. NINFEA

NINFEA is an internet-based birth cohort study recruiting pregnant women, started in 2005 in the city of Turin and then extended to the rest of Italy (www.progettoninfea.it) [20]. For this paper we used the NINFEA database version 09.2018 that consists of 6625 mothers and 7423 pregnancies.

Data on dwelling type, house size (m^2^), family size, maternal cohabitation status, age, education, country of birth, occupational status, jobs code according to the ISCO-88, and on paternal education, occupational status and mother tongue are available for the baseline period. As for Piccolipiù, the value of the geographical-based deprivation index has been assigned to each NINFEA participant on the basis of the address of residence at recruitment.

Child weight and height data, used to derive the BMI, are collected at each follow-up questionnaire. For this paper we used the 18-month and 4-year measures.

#### 2.1.4. ELFE

ELFE is a French national birth cohort, that consists of 18,040 mothers and 18,329 babies born in 2011 [21].

The parental and household social data analyzed in this study were collected at the 2-months telephone survey. These include: dwelling type, tenure status, number of rooms, household size, maternal cohabitation status, age, education, country of birth and occupational status (coded according to the French Profession et social category and converted into ISCO-88 codes). Total household gross income was collected as well as perceived financial hardship and bank overdraft frequency over the last year. Weight and height were reported by the interviewed parent. Predicted weight and height at 2 years of age were calculated using previously modelled trajectories from the Jenss–Bayley model [22], and were used to derive the predicted BMI.

#### 2.1.5. EDEN

The EDEN mother-child cohort study was designed to assess pre- and post-natal determinants of child growth, development and health [23]. In brief, between 2003 and 2006, 2002 pregnant women (<24 gestational weeks) aged 18–45 year were recruited at Nancy and Poitiers university hospitals.

Parental and household social data were collected during pregnancy (24–28 gestational weeks) or at delivery and included: dwelling type, tenure status, number of rooms, household size, maternal cohabitation status, age, education, country of birth, occupational status, ISCO-88 job codes, and on paternal age, education, country of birth, occupational status and ISCO-88 job codes. The mother also reported net household income, perceived financial hardship (ranging 0 to 3) and bank overdraft frequency over the last year.

Weight and height were measured by previously trained midwives at birth, 1, 3, and 5 years. Additionally, mothers filled in self-administered questionnaires at 4 months, 8 months and 1, 2, 3, 4 and 5 years where they reported measured growth data available in their child’s health booklet. Using all available collected data, predicted weight and height at 2 and 4 years were calculated using previously modelled trajectories from the Jenss–Bayley model [22].

### 2.2. The Equivalized Household Income Indicator (EHII)

Among the income measures available in EUSILC, we selected the total disposable household income, which is the sum of the gross personal income components of all household members and the gross income components at household level minus regular taxes on wealth, regular inter-household cash transfer paid and tax on income and social insurance contributions [16]. The personal income components include gross employee cash or near cash income, company car, gross cash benefits or losses from self-employment—including royalties, pensions received from individual private plans, benefits for unemployment, old-age, survivor, sickness and disability, and education-related allowances. The gross income components at household level include income from rental of a property or land, family/children related allowance, housing allowances, regular inter-household cash transfers received, interests/dividends/profit from capital investments in unincorporated business and income received by people aged under 16. In order to account for differences in the household size and composition, we derived the equivalized income as the ratio between the total disposable household income and the equivalized household size. The latter is available in the EUSILC database and is calculated as the sum of the weights given to all the members of the household: 1 to the first adult; 0.5 to the second and each subsequent person aged 14 and over; and 0.3 to each child aged under 14 [24].

We derived the cohort-specific EHII according to the following steps (which are further explained below): (i) identification of the potential predictors of the equivalized household disposable income available both in the country-specific EUSILC database and in the specific cohort; (ii) selection of the EUSILC analysis samples to develop and validate the prediction model (iii) construction of the prediction model; (iv) evaluation of the model performance. The regression coefficients obtained from the prediction model were then applied to the cohort data to derive the EUSILC-based income indicator.

The prediction models are cohort- and period-specific as they depend on the information available in the cohort at the different time points. In this paper we derived the income indicator for the baseline period, i.e., before or during pregnancy or at birth depending on the cohort.

#### 2.2.1. Predictors

We selected as potential predictors the EUSILC household and personal variables likely to be available in birth/pregnancy cohorts. The personal data included age, educational level, occupational status, ISCO code, country of birth, marital status and cohabitation status (living with/without a partner); while the household variables were dwelling type, tenure status, number of rooms, and family size. Until 2011, EUSILC coded jobs using the first 2 digits of the ISCO-88 classification, while the first 2 digits of the ISCO-08 classification were used from 2011 onwards. For the 2011 survey both versions are available. For this study we used the 1-digit variables only.

#### 2.2.2. Analysis Sample

The model for each cohort was constructed using the EUSILC data of the country of the cohort (i.e., Italy-EUSILC survey data for Piccolipiù and NINFEA, French-EUSILC survey data for ELFE and EDEN). The household was the unit of analysis; all households including at least one child (16-years old or younger) and his/her mother were included. Households with 8 or more members, households with errors in the id variables and households with very atypical/rare family structure (e.g., two or more family units living together) were excluded (0.2% in both 2011 Italian and French database). Moreover, household with an equivalized total disposable income below/above the lower/upper limits, where the lower limit is Q1−1.5×I IQR and the upper limit is Q3+1.5×IQR (Q1 and Q3 indicates the 25th and 75th percentiles respectively and IQR the interquartile range) were excluded (about 5% and 3% of the Italian and French samples respectively). In EUSILC, for each household member, the identification code of his/her father, mother and partner are available if they live in the same household. This identification code was used to link personal data of the mother and, if present, of the father with the household data for each selected household.

Due to the EUSILC sampling frame and sample selection methods, a non-zero probability of selection is assigned to every individual and household in the target population. To account for this sampling scheme, household weights were taken into account in the statistical analyses.

For the development of the prediction model we used the 2011 survey data, because in that survey jobs were coded using both ISCO-88 and ISCO-08 codes. In Italy and France, as well as in most of the other countries, the survey has been conducted using a rotational design, with one-quarter of the sample rotating from one year to the next; for this reason, we used as a validation set the data from 2015 (temporal validation), which are completely independent from those from 2011. In order to validate the models, we used the ISCO-08 codes in both the developmental and validation sets, but we used the ISCO-88 codes when we estimated—in the 2011 data—the coefficients to derive the predicted index, as most birth cohorts in Europe coded jobs using the ISCO-88 classification.

#### 2.2.3. Model Building

The equivalized total disposable household income has a severely positively skewed distribution (p-values from the skewness/kurtosis test for normality in the French and Italian analysis sample < 0.0001). Therefore, we used multivariable linear regression models with log-transformation of the outcome. For each cohort, we identified the EUSILC variables available in the cohort for at least 90% of the subjects to be used as predictors. These variables were formatted in EUSILC to match the categorization available in the cohort. To avoid missing values by design, inactive subjects, who do not have, by definition, an ISCO code for the occupation were assigned to the most frequent ISCO class. The same approach was used for the paternal variables for the households with a single mother. Different shapes of the relationship of the continuous variables with the outcome were evaluated. Prediction models were performed using a complete case analysis approach.

#### 2.2.4. Model Performance

The overall model performance was assessed based on the value of the R^2^ statistics, that was calculated both for the 2011 and the 2015 model. Calibration was examined using the calibration plots (scatter plot of the observed outcomes by decile of the predicted outcomes) and the calibration slope, where the latter reflects the combined effect of overfitting on the development data and true temporal differences in the coefficients.

#### 2.2.5. Derivation of the EHII in Each Cohort

To derive the EHII for each cohort member, the regression coefficients obtained in the developmental data were applied to the individual cohort data. As the focus is typically on the rank rather than on the absolute value of the income, in particular for studies using data from different countries, we categorized the predicted log-transformed EHII using the quintiles as the cut-offs. To obtain the value of the EHII on the original scale accounting for non-linearity in the log-transformation we back-transformed it using the following approach: (i) we added to the predicted income (log-euro) a draw from the estimated distribution of the error term, for each individual, and then exponentiate it; (ii) we repeated this step 100 times; (iii) we took the average of the 100 mean values. Absolute values should be interpreted as the equivalized total disposable household income a family with those specific characteristics would have had in 2011.

#### 2.2.6. Analysis of the EHII

We described the distribution of the available predictors and of other SEP-related variables within each cohort-specific predicted EHII quintile. Moreover, we estimated the association between the EHII in quintiles and continuous BMI at 2 and 4 years of age in the four cohorts using linear regression models.

## 3. Results

Although with different level of detail, the following predictors were available in all four birth cohorts analyzed: maternal age, cohabitation status, country of birth, educational level, occupational status and occupational code; paternal/partner country of birth, educational level and occupational status; and household size. Moreover, paternal/partner age and occupational code, and household tenure status were available in all cohorts except NINFEA; dwelling type was available in all cohorts except Piccolipiù; and maternal marital status was available in the French cohorts only.

The value of the R^2^ statistic obtained when fitting the model in the developmental data (2011 surveys) reflected the amount of data available, being equal to 0.45, 0.41, 0.53 and 0.51, for the Piccolipiù, NINFEA, ELFE and EDEN cohorts respectively. When the models were validated using the 2015 data, the values of the R^2^ statistic decreased slightly to 0.42, 0.39, 0.52 and 0.51, while the calibration slopes were equal to 0.96, 0.96, 1.01 and 0.98 respectively, indicating a good temporal validation. The Supplementary Figures S1–S4 show the calibration plots, that is the scatter plot of the mean observed log-income vs. mean predicted log-income by decile of the predicted outcome. Supplementary Tables S1–S4 report the coefficients obtained from the four models. The paternal/partner country of birth was not included in the Piccolipiù and NINFEA models as this variable had no impact on the prediction capability and was missing for approximately 4% of the subjects in each cohort, while the dwelling type was not included in the EDEN model due to a large amount of missing data. In all models parental age was included as a continuous variable.

The directions and magnitudes of the coefficients of the single predictors were consistent across the four studies; living with a partner, being born in the country of the cohort, having a higher education, being employed/self-employed, owning the house, living in a bigger house and having a lower household size were positively associated with the EHII. These data are reflected in the results shown in Table 1, where the cohort-specific quintiles of the EHII are described in terms of the available predictors: among those predicted to have the highest income there are no households with a single mother, or with an unemployed parents in all cohort, the majority (from 86% to 98%) have parents with a post-secondary education or higher, and almost (from 94% to 100%) all have parents born in the country of the cohort (Table 1). The variables excluded from the prediction models because of missing values (i.e., paternal country of birth for the Italian cohorts and dwelling type in EDEN) are included in this table.

The mean values of the EHII back-transformed in Euros are equal to 1758, 1807, 1895, and 1725 € in the Piccolipiù, NINFEA, ELFE and EDEN cohorts respectively.

**Figure 1:**
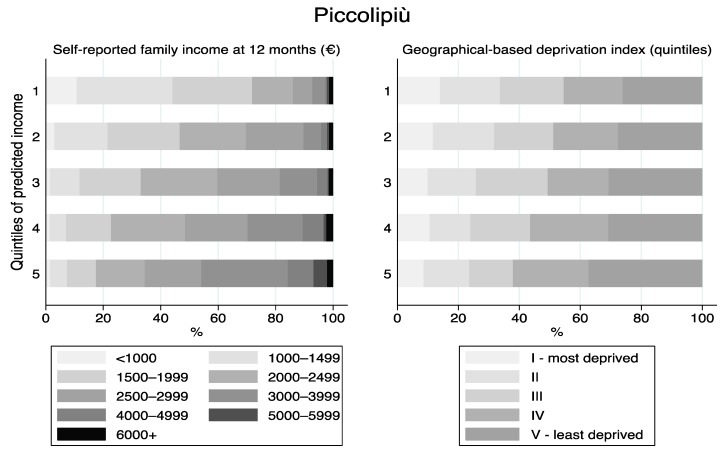
Distribution of the socioeconomic position (SEP)-related variables available in the Piccolipiù cohort by quintiles of the predicted income.

**Figure 2:**
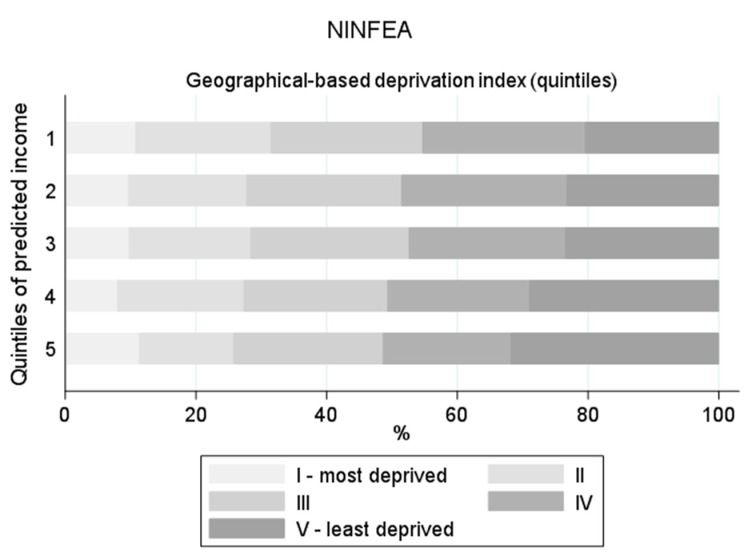
Distribution of the SEP-related variables available in the NINFEA cohort by quintiles of the predicted income.

**Figure 3:**
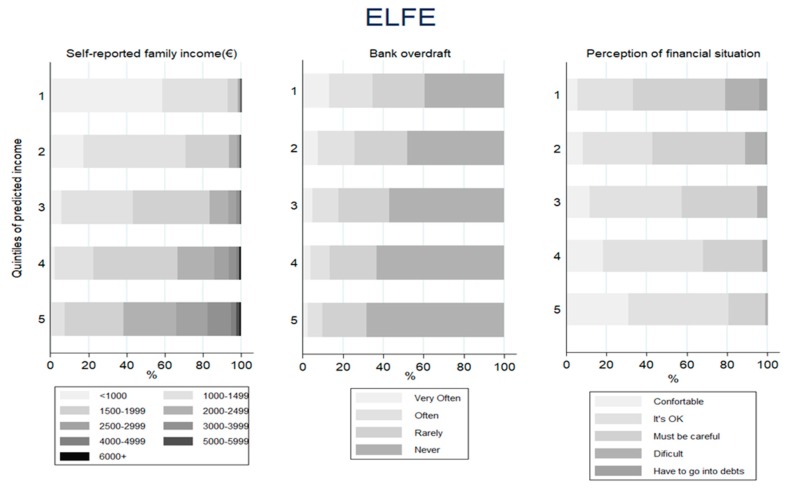
Distribution of the SEP-related variables available in the ELFE cohort by quintiles of the predicted income.

**Figure 4:**
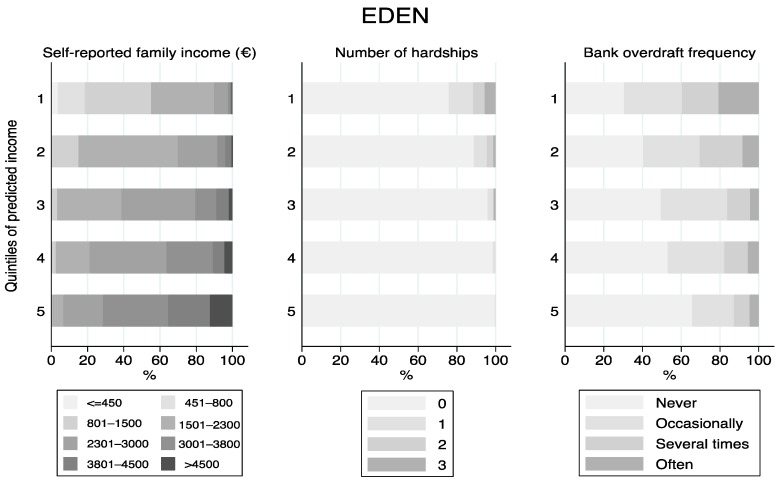
Distribution of the SEP-related variables available in the EDEN cohort by quintiles of the predicted income.

Figure 1, Figure 2, Figure 3 and Figure 4 displays the distribution of the predicted quintiles in terms of the other available cohort-specific SEP-related variables: self-reported monthly net family income at 12 months and geographical deprivation index in Piccolipiù (Figure 1); geographical deprivation index in NINFEA (Figure 2); self-reported income at 2 months, bank overdraft and perception of financial situation in ELFE (Figure 3); and self-reported income at recruitment, bank overdraft and number of hardships in EDEN (Figure 4). In all cohorts, and in particular in the French studies, there was a strong correlation between the self-reported income, as collected by questionnaires, and the EHII. There was a clear association also between the other individual SEP-related variables available in the French cohorts and the EHII: for example, the proportion of those reporting to have experienced a bank overdraft often or several times over the last 12 months in EDEN was 39% among those with the lowest predicted income and about 13% among those in the highest quintile (Figure 4), while the corresponding proportions of those answering no bank overdraft were approximately 30% vs. 65%, with very similar results in the ELFE cohort (Figure 3). Consistent findings were observed when analysing the “perceived financial situation” variable in ELFE and the “number of financial hardships” variable in EDEN. The association between the EHII and the geographical deprivation index, available in the Italian cohorts, was weaker. In Piccolipiù 26% of those predicted to have the lowest income were resident in the least deprived area according to the geographical index compared with 38% among those predicted to have the highest income (Figure 1). The corresponding figures in NINFEA were 20% vs. 30% (Figure 2).

Finally, Table 2 shows the estimates of the crude associations of maternal education (categorized in three levels) and of the EHII (in quintiles) with child BMI at two and four years of age separately in each cohort (BMI at four years of age is not available in ELFE, while BMI at 18 months and not at 24 months is available in NINFEA). In all cohorts we observed an inverse association of the EHII with BMI at both two and four years of age, with the exception of NINFEA at 18 months; additionally, maternal education was inversely associated, but with less consistent results across the cohorts. Adjustment for maternal education did not affect the associations between the EHII and BMI at two years, while slightly attenuated the effects at 4 years of age (data not shown).

## 4. Discussion

This paper describes a method for constructing a new standardized and comparable household income indicator (EHII) for child SEP to be used in birth cohort studies. The method is applied in four birth cohorts from two countries, Italy and France, and the derived EHII is described comparing its distribution with that of other SEP-related variables and estimating the cohort-specific associations between the EHII and infant and childhood BMI. The paper shows that using basic parental and household characteristics, typically available in birth cohort studies, it is possible to predict the household income with a fairly good prediction model performance (R^2^ ranging between 0.41 and 0.53). The models were validated and the directions and magnitudes of the coefficients of the single predictors were consistent across the four studies. There was also a strong correlation between the predicted income and both the self-reported income, as collected by questionnaires, and the other individual SEP-related variables available (bank overdraft, perception of financial situation and number of hardships). The association between the EHII and the geographical deprivation index, available in the Italian cohorts only, was weaker. Finally, in all cohorts we observed an inverse association between the EHII quintiles and BMI, an outcome known to be strongly socially shaped [25].

The proposed method has some limitations. First, the models being cohort-specific, as they depend on the availability of the predictors in each cohort, model misspecification varies across the different studies. Furthermore, in its current version, we are not accounting for the prediction model error. Finally, being based on EUSILC this indicator cannot be used in non-European studies, although the approach can be applied to all countries where a survey/database similar to EUSILC exist.

The proposed EHII has several implications for epidemiological studies: (i) it allows to have a standardized and comparable child SEP indicator over different studies, (ii) it can be derived for all studies that are based in those European countries (*n* = 31) that are included in the EUSILC survey; (iii) it gives a measure of the household income, a domain which is otherwise very difficult to assess through questionnaires; (iv) it captures a SEP dimension different from and complementary to the one captured by the educational level.

Being based on external data from the EUSILC surveys, which are conducted in several European countries using the same design and procedures, the EHII allows obtaining a harmonized family income measure over different European populations. This is an essential need in the context of international collaborative studies. Other cross-country comparable composite SEP indicators have been proposed in the epidemiological literature, although none is focused on the household income. Among these the European Socio-Economic Classification is an occupational based index used as a SEP indicator in the H2020 LIFEPATH project [13]; the European Deprivation Index [26] is an ecological indicator constructed from the EUSILC survey and therefore in principle applicable to all European studies, even if the neighborhood/ecological deprivation likely affects health outcome through different mechanisms than the individual SEP. The household disposable income is one of the most important individual single indicators of child SEP, but is difficult to obtain through questionnaires; for example, in this study household income was available in all cohorts except NINFEA, but only in ELFE was assessed thoroughly. It follows that, mainly because of feasibility issues, epidemiological studies involving several birth cohorts typically use maternal education as the only indicator of SEP. Maternal education however might be insufficient to capture the multidimensionality of health inequalities or to control for confounding when SEP is an important potential confounder. Moreover, maternal education is practically stable over time, and, even when it changes, it may only increase, while the EHII is expected to vary over time and can capture longitudinal variations in SEP. The fact that the EHII is sensitive to longitudinal changes is of particular importance when studying the potential impact of economic crises that can hit strata of the population differently. Finally, the EHII can be used not only to measure child SEP within the framework of birth cohort research, but could contribute also to other epidemiological areas, as, for example, when it comes to population health surveys or adult cohorts.

## 5. Conclusions

The development of the equivalized household income indicator, contributes to improving the research on social inequalities in health, in particular in the context of European birth cohort collaborative studies, where it is essential to have harmonized comparable SEP indicators over the different studies.

## Figures and Tables

**Table 1:** Description of the cohort-specific predicted income quintiles in terms of the available predictors.

	Cohort-Specific Quintiles of the Predicted Equivalized Total Disposable Household Income																			
	Piccolipiù (*n* = 3105)					NINFEA (*n* = 6980)					ELFE (*n* = 13,544)					EDEN (*n* = 1815)				
	1	2	3	4	5	1	2	3	4	5	1	2	3	4	5	1	2	3	4	5
	*%* ^a^					*%* ^a^					*%* ^a^					*%* ^a^				
**Household**																				
Dwelling type ^b^																				
Detached	--	--	--	--	--	24.5	21.0	16.0	15.3	9.8	40.1	59.5	66.0	67.1	58.9	51.6	69.3	76.8	78	78.3
Semi-detached	--	--	--	--	--	15.0	16.3	16.6	15.6	14.4	5.4	1.4	1.3	1.4	1.5	30.0	21.8	20.3	19.8	18.3
Flat	--	--	--	--	--	56.2	61.0	66.3	68.3	75.1	54.5	39.1	32.7	31.5	39.6	18.4	8.9	2.94	2.26	3.43
Other kind	--	--	--	--	--	4.3	1.7	1.1	0.8	0.7	--	--	--	--	--	--	--	--	--	--
Tenure status																				
Owner	42.8	64.1	76.3	82.3	94.2	--	--	--	--	--	13.4	42.8	60.5	71.8	81.3	11.3	33.3	51.2	60.1	79.1
Tenant	49.9	27.7	18.1	15.8	4.2	--	--	--	--	--	41.3	38.2	28.3	21.4	15.6	77.1	63.4	45.5	37.5	20.1
Tenant reduced rate	--	--	--	--	--	--	--	--	--	--	40.7	13.7	5.8	2.1	0.7	--	--	--	--	--
House for free	7.3	8.2	5.6	1.9	1.6	--	--	--	--	--	4.6	5.3	4.4	4.7	2.4	11.6	3.3	3.3	2.5	0.8
Number of rooms ^c^																				
1–2	26.4	18.5	16.3	15.5	10.0	8.2	6.5	4.9	4.4	2.8	12.7	6.4	3.9	3.6	1.8	15.7	6.9	5.5	1.4	1.1
3	34.8	39.0	35.8	34.6	34.0	62.3	60.6	64.0	59.4	54.2	29.3	23.9	21.3	17.2	18.0	36.1	32.8	24.8	23.1	14.3
4	23.2	24.8	30.1	28.0	27.0	19.5	20.4	20.5	22.3	22.8	27.3	27.2	27.6	24.7	25.8	28.4	32.8	34.4	29.5	25.3
5	9.2	10.8	11.4	14.8	19.3	10.0	12.5	10.6	13.9	20.2	17.1	22.5	23.5	26.4	25.4	10.4	16.5	20.1	26.7	26.5
≥6	6.4	6.9	6.4	7.1	9.7	--	--	--	--	--	13.6	20.0	23.7	28.1	29.0	9.4	11.0	15.2	19.3	32.8
Household size																				
2	1.0	2.3	0.2	0.0	0.0	28.9	8.6	0.1	0.0	0.0	6.1	2.8	0.6	0.0	0.0	7.2	4.4	3.3	1.1	3.9
3	33.0	48.3	58.9	62.3	68.0	25.9	49.1	64.5	63.9	79.8	29.3	38.5	40.7	46.1	53.1	31.1	37.7	44.6	46.6	51.2
4	37.7	37.0	32.5	31.4	28.6	23.9	26.6	28.0	27.1	18.0	30.6	37.9	40.9	40.2	33.9	27.8	40.5	36.7	36.6	34.5
5	16.4	9.3	7.1	5.0	2.6	11.8	9.9	5.2	6.7	1.9	19.9	15.5	14.3	11.1	10.7	22.3	12.4	9.9	12.7	8.5
≥6	11.9	3.1	1.3	1.3	0.8	9.5	5.8	2.2	2.3	0.3	14.1	5.3	3.5	2.6	2.3	11.6	5.0	5.5	3.0	1.9
**Maternal**																				
Single mothers	8.2	4.5	0.2	0.0	0.0	37.8	9.0	0.0	0.0	0.0	22.1	4.0	0.8	0.0	0.0	23.4	5.23	1.38	0.0	0.0
Separated/Divorced/Widow	--	--	--	--	--	--	--	--	--	--	4.9	3.3	2.0	1.2	1.6	3.0	3.6	2.2	0.8	2.8
Country of birth																				
Italy/France	77.9	91.3	94.9	96.1	98.9	88.5	96.4	96.4	98.4	99.9	77.9	90.9	93.6	94.4	94.3	90.9	95.9	96.4	97.2	97.8
Other EU	13.2	5.6	3.5	3.1	0.5	7.9	2.2	2.6	1.2	0.1	1.4	2.2	2.6	2.6	3.5	0.0	1.1	0.6	0.6	1.4
Other	8.9	3.1	1.6	0.8	0.6	3.6	1.4	1.0	0.4	0.0	20.7	6.9	3.8	3.0	2.2	9.1	3.0	3.0	2.2	0.8
Education																				
≤primary	1.3	0.0	0.0	0.0	0.0	0.9	0.1	0.0	0.0	0.0	9.4	1.4	0.2	0.1	0.0	25.1	6.6	2.4	0.6	0.0
lower secondary	33.7	15.9	5.5	1.0	0.3	18.1	6.4	1.7	0.5	0.1	50.6	24.3	5.5	1.4	0.2	46.0	39.9	18.2	1.9	0.6
upper secondary	51.0	63.3	56.2	33.6	12.4	50.0	52.9	42.8	16.5	5.0	29.4	36.1	20.3	7.8	1.9	24.0	28.2	27.3	10.2	0.8
≥post-secondary	14.0	20.8	38.3	65.4	87.3	31.0	40.7	55.5	83.0	94.9	10.6	38.2	74.0	90.7	97.9	4.9	25.3	52.1	87.3	98.6
Occupation ^d^																				
employed	40.7	76.5	80.7	80.7	82.9	44.1	72.1	83.9	83.2	85.9	31.8	69.6	83.6	90.3	93.4	21.2	79.1	85.7	94.8	100
self-employed	7.3	11.6	15.0	17.7	17.1	10.2	13.3	12.8	16.6	14.1	1.7	3.5	5.1	6.9	6.6	--	--	--	--	--
unemployed	19.3	5.5	2.4	0.6	0.0	19.4	7.6	0.4	0.1	0.0	29.0	18.0	7.7	2.3	0.0	32.5	11.6	7.44	2.48	0.0
domestic task	29.5	3.5	0.3	0.0	0.0	17.8	1.7	0.0	0.0	0.0	34.0	7.6	3.1	0.4	0.0	31.7	3.86	4.68	1.38	0.0
other	3.2	2.9	1.6	1.0	0.0	8.5	5.3	2.9	0.1	0.0	3.5	1.3	0.5	0.1	0.0	14.6	5.51	2.2	1.38	0.0
ISCO88 ^e^																				
0	0.0	0.0	0.2	0.2	0.0	0.0	0.0	0.1	0.0	0.0	--	--	--	--	--	--	--	--	--	--
1	7.4	11.0	10.3	10.6	5.0	10.7	8.9	6.2	7.5	3.4	0.0	1.3	3.0	6.8	18.2	0.0	0.6	1.1	3.9	4.9
2	4.4	11.3	21.6	36.3	54.8	17.4	20.8	27.5	42.7	53.9	0.8	8.6	25.7	42.7	56.1	0.0	1.9	4.4	11.3	30.0
3	6.0	9.0	11.8	16.2	15.0	7.4	8.5	10.6	11.1	9.4	68.7	37.5	33.9	29.5	17.4	24.5	24.0	42.7	62.0	61.2
4	9.7	24.2	34.9	30.8	24.6	26.4	41.5	50.3	36.5	33.2	4.6	16.8	24.4	16.1	7.6	20.4	32.5	38.3	17.9	3.6
5	48.3	37.1	18.4	4.9	0.6	23.2	15.5	4.1	1.7	0.0	13.1	19.6	8.2	3.1	0.3	38.8	32.5	11.0	3.6	0.3
6	0.3	0.0	0.3	0.0	0.0	0.3	0.2	0.2	0.0	0.0	0.3	0.8	0.2	0.2	0.0	0.3	0.3	0.3	0.5	0.0
7	2.4	2.2	1.2	0.5	0.0	2.9	1.9	0.4	0.4	0.1	4.4	5.7	2.4	0.8	0.3	6.3	5.5	1.4	0.8	0.0
8	3.0	2.6	1.5	0.5	0.0	1.7	1.6	0.4	0.0	0.0	0.8	1.1	0.4	0.3	0.0	2.2	1.6	0.5	0.0	0.0
9	18.5	2.6	0.0	0.0	0.0	10.0	1.1	0.2	0.1	0.0	7.3	8.6	1.8	0.5	0.1	7.5	1.1	0.3	0.0	0.0
Age (mean)	31.5	33.6	34.1	34.9	35.8	31.3	32.8	33.5	34.2	34.8	28.8	30.4	31.4	31.9	33.0	26.8	29.2	30.2	30.7	31.2
**Paternal**																				
Country of birth ^f^																				
Italy/France	79.8	95.5	97.5	96.2	96.3	91.5	96.9	97.9	97.6	97.8	80.0	90.5	93.6	94.8	95.5	89.5	92.0	95.6	93.9	95.0
Other EU	11.9	2.9	0.7	2.8	2.6	8.5	3.1	2.1	2.4	2.2	1.8	1.8	1.7	1.6	2.4	0.6	1.9	1.1	0.3	2.2
Other	8.3	1.6	1.8	1.0	1.1	--	--	--	--	--	18.2	7.7	4.7	3.6	2.1	9.9	6.1	3.3	5.8	2.8
Education																				
≤primary	2.1	1.1	0.2	0.0	0.0	2.9	0.6	0.0	0.0	0.0	8.1	2.7	1.5	0.1	0.0	17.1	14.3	7.7	3.6	0.8
lower secondary	47.5	34.6	20.0	3.2	0.6	37.6	34.2	12.7	0.2	0.0	35.8	34.1	23.6	10.7	1.6	36.6	39.7	26.4	19.3	3.3
upper secondary	44.1	52.0	66.1	55.6	10.7	44.0	46.6	74.5	47.6	1.6	46.2	36.8	31.6	13.4	2.8	39.7	28.1	29.5	16.5	3.1
≥post-secondary	6.3	12.3	13.7	41.2	88.7	15.5	18.6	12.8	52.2	98.4	9.9	26.4	43.3	72.8	95.6	6.6	17.9	36.4	60.6	92.8
Occupation ^d^																				
employed	54.9	63.3	71.0	77.5	80.0	85.3	93.7	98.5	98.7	99.9	71.6	79.6	82.5	85.7	95.3	81.8	92.6	94.8	98.3	100
self-employed	31.1	34.1	28.7	22.2	20.0	--	--	--	--	--	8.6	14.4	13.5	12.7	4.7	--	--	--	--	--
unemployed	13.2	1.8	0.0	0.0	0.0	9.5	2.6	0.1	0.0	0.0	16.8	5.0	3.0	0.6	0.0	14.0	3.3	2.7	1.1	0.0
other	0.8	0.8	0.3	0.3	0.0	5.2	3.7	1.4	1.3	0.1	3.0	1.0	1.0	0.0	0.0	4.2	4.1	2.5	0.6	0.0
ISCO88 ^e^																				
0	2.1	0.7	1.8	2.3	1.8	--	--	--	--	--	--	--	--	--	--	--	--	--	--	--
1	14.2	15.4	16.8	19.1	13.4	--	--	--	--	--	1.1	3.6	6.5	11.0	22.4	0.3	0.8	2.5	2.5	10.7
2	3.2	6.5	9.1	19.5	52.6	--	--	--	--	--	0.9	4.7	11.4	22.5	64.4	0.3	0.8	6.1	9.1	42.1
3	6.7	11.1	19.1	20.5	13.4	--	--	--	--	--	53.2	33.0	33.0	35.7	8.9	36.1	20.7	35.0	49.5	39.4
4	6.6	11.7	14.7	23.2	15.8	--	--	--	--	--	2.8	5.4	7.6	7.5	2.1	10.5	16.0	18.2	15.2	4.7
5	15.0	17.2	11.0	4.4	1.0	--	--	--	--	--	5.5	7.7	7.5	4.3	0.6	4.1	7.5	4.7	6.9	1.1
6	1.7	1.0	1.0	0.3	0.2	--	--	--	--	--	2.0	3.2	1.5	0.1	0.0	0.5	5.2	3.0	0.0	0.0
7	27.2	22.5	13.1	3.6	0.0	--	--	--	--	--	21.9	27.2	21.3	12.0	1.2	34.4	35.8	22.0	14.3	1.4
8	10.7	8.1	11.0	6.6	1.8	--	--	--	--	--	6.5	6.9	5.8	3.2	0.1	6.6	6.6	4.1	1.4	0.0
9	12.6	5.8	2.4	0.5	0.0	--	--	--	--	--	6.1	8.3	5.4	3.7	0.3	7.2	6.6	4.4	1.1	0.6
Age (mean)	35.0	36.7	37.0	37.7	38.2	--	--	--	--	--	34.1	32.7	33.4	33.6	34.4	30.5	31.7	32.0	32.8	33.3

^a^ Colum percentage; ^b^ In ELFE and EDEN detached = house, semi-detached = flat in a big building, flat = flat in a small building; ^c^ In NINFEA the last class is ≥5; ^d^ In EDEN the employed and self-employed classes are combined; ^e^ In ELFE and EDEN class 0 (Armed forces) is not available, in EDEN classes 1 (Legislators, senior officials and managers) and 2 (Professionals) are combined; ^f^ In NINFEA the paternal country of birth information is based on paternal mother tongue and two classes are available: Italian vs. Other.

**Table 2:** Associations between predicted income (quintiles) and child BMI at 2 and 4 years of age by cohort.

	Piccolipiù (*n* = 1675) ^a^			NINFEA (*n* = 4530) ^a,b^			ELFE (*n* = 12,069)			EDEN (*n* = 1685)		
	*Coef*	*95% CI*	*p-Trend*	*Coef*	*95% CI*	*p-Trend*	*Coef*	*95% CI*	*p-Trend*	*Coef*	*95% CI*	*p-Trend*
	**BMI at 2 Years of Age**											
Maternal education												
≤lower secondary	−0.08	−0.35; 0.20	0.01	−0.13	−0.38; 0.12	0.24	0.05	−0.03; 0.13	0.03	0.04	−0.12; 0.21	0.08
secondary	--			--			--			--		
≥post-secondary	−0.24	−0.39; −0.08		0.03	−0.08; 0.13		−0.06	−0.12; 0.01		−0.07	−0.22; 0.08	
Predicted income												
1	--		0.001	--		0.84	--		<0.001	--		0.013
2	−0.19	−0.45; 0.06		0.01	−0.15; 0.18		−0.08	−0.16; 0.00		−0.05	−0.23; 0.13	
3	−0.15	−0.40; 0.10		−0.13	−0.29; 0.03		−0.13	−0.21; −0.05		−0.15	−0.33; 0.02	
4	−0.29	−0.53; −0.04		0.02	−0.13; 0.18		−0.16	−0.24; −0.08		−0.15	−0.33; 0.02	
5	−0.43	−0.68; −0.19		0.01	−0.15; 0,17		−0.18	−0.26; −0.10		−0.20	−0.37; −0.02	
	**BMI at 4 Years of Age**											
Maternal education												
≤lower secondary	0.25	−0.04; 0.55	0.11	0.10	−0.19; 0.40	0.01				0.01	−0.16; 0.18	0.012
secondary	--			--						--		
≥post-secondary	−0.03	−0.21; 0.14		−0.15	−0.27; −0.03					−0.15	−0.3; 0	
Predicted income												
1	--		0.05	--		0.03				--		0.001
2	−0.14	−0.42; 0.15		−0.24	−0.42; −0.05					0.02	−0.16; 0.21	
3	−0.08	−0.36; 0.19		−0.22	−0.40; −0.04					−0.11	−0.29; 0.07	
4	−0.17	−0.45; 0.10		−0.18	−0.36; 0.00					−0.15	−0.33; 0.03	
5	−0.27	−0.54; −0.00		−0.26	−0.44; −0.09					−0.25	−0.43; −0.07	

^a^ The sample size for the analysis at 4 years of age decreases to 1237 in Piccolipiù and 3761 in NINFEA; ^b^ In NINFEA weight and height data are available at 18 months and not at 24 months.

## Supplementary Materials

The following are available online at https://www.mdpi.com/1660-4601/17/5/1700/s1, Figure S1: Calibration plot for the Piccolipiù model, Figure S2: Calibration plot for the NINFEA model, Figure S3: Calibration plot for the ELFE model, Figure S4: Calibration plot for the EDEN model, Table S1: Piccolipiù prediction model, Table S2: NINFEA prediction model, Table S3: ELFE prediction model, Table S4: EDEN prediction model.
