# Supplementary material for: Measuring Child Socio-Economic Position in Birth Cohort Research: The Development of a Novel Standardized Household Income Indicator
Source: Int J Environ Res Public Health. 2020 Mar 5;17(5):1700. doi: 10.3390/ijerph17051700 (PMC7084936; doi:10.3390/ijerph17051700)
Supplement: Supplementary file 1 [file ijerph-17-01700-s001.pdf]

Supplementary Material to

Measuring child socio-economic position in birth cohort research: the development of a novel standardized household income indicator

Table of contents

SUPPLEMENTARY METHODS

EUSILC database

Cohort specific study population description

SUPPLEMENTARY TABLES

Table S1 Piccolipiù prediction model

Table S2 NINFEA prediction model

Table S3 ELFE prediction model

Table S4 EDEN prediction model

SUPPLEMENTARY FIGURES

Fig. S1 Calibration plot for the Piccolipiù prediction model

Fig. S2 Calibration plot for the NINFEA prediction model

Fig. S3 Calibration plot for the ELFE prediction model

Fig. S4 Calibration plot for the EDEN prediction model

Supplementary Methods

EUSILC database

The European Union Statistics on Income and Living Conditions (EUSILC) is a survey that collects from 2005 and onwards comparable annual microdata at both individual (basic demographic data, education information, limited health data, labour force data, income data) and household level (housing conditions, basic demographic data, material deprivation and aggregated income data) in representative samples of persons aged at least 16 years in 28 European Union States - as well as Iceland, Norway and Switzerland (~500,000 European residents annually). Individual data can be linked to household data and viceversa. EUSILC has both a cross-sectional and a longitudinal component, but for this study we used the cross-sectional data only. Depending on the country, microdata could come from: i) two or more national sources (surveys and/or registers); ii) one or more existing national sources combined or not with a new survey; iii) a new harmonized survey. For all components of EUSILC (whether survey- or register-based), both the cross-sectional and longitudinal initial sample data are based on a nationally representative probability sample of the population residing in private households within the country.

For this study we used the 2011 and 2015 Italian and French data. The sample size of the 2011 Italian and French surveys was of 19,399 and 11,360 households respectively. Of these 4,706 and 3,071 respectively met the inclusion criteria and were used to derive the coefficients.

Cohort specific study population description

Piccolipiù

Piccolipiù is an Italian multicentre cohort, involving 5 centers (Turin, Trieste, Firenze, Viareggio, and Roma) that have recruited from 2011 to 2015 about 500 newborns each (1000 in Roma) for a total of approximately 3400 newborns. Singleton pregnant women were eligible if they were aged at least 18 years, resident in the catchment area of the selected hospitals, and have enough knowledge of the Italian language. Mothers completed multi-purpose questionnaires at recruitment (around delivery), and when the child turned 6 months and 1, 2, 4 and 6 years. Women also completed diaries during the first three years to record anthropometric measurements.

The study has been approved by the Ethics committees of the Local Health Unit Roma E (coordinating centre), of the Istituto Superiore di Sanità (National Institute of Public Health) and by the Ethics committees of each local centre.

NINFEA

NINFEA (Nascita ed INFanzia: gli Effetti dell'Ambiente) is an internet-based birth cohort study recruiting pregnant women, started in 2005 in the city of Turin and then extended to the rest of Italy. Recruitment has been carried out in the period 2005–2016, with the study advertised both actively, through obstetrics clinics, and passively, via Internet and the media. Members of the cohort are children of mothers who have access to the Internet, have enough knowledge of the Italian language to complete on-line questionnaires and volunteer to participate any time during pregnancy completing the first baseline questionnaire. Children are followed up by six additional questionnaires completed by the mothers 6 and 18 months after delivery and when children turn 4, 7, 10 and 13 years of age.

The Ethical Committee of the San Giovanni Battista Hospital and CTO/CRF/Maria Adelaide Hospital of Turin approved the NINFEA study (approval N. 0048362, and subsequent amendments) and the informed consent was obtained from all the participants.

ELFE

ELFE (Étude Longitudinale Française depuis l'Enfance) is a French national birth cohort, that consists of 18040 mothers and 18329 babies born in 2011. Children were recruited at birth in 2011 from 349 randomly selected maternity units in metropolitan France. Inclusion criteria were: single or twin live births at ≥ 33 weeks of gestation; mother aged ≥ 18 years, no plan to leave metropolitan France within 3 years. Information and consent documents were provided in French, Arabic, Turkish and English. More than 96% of mothers who fulfilled the first two inclusion criteria (n=37 494) were contacted by research assistants during their stay at the maternity and 51% (18040) agreed to participate in the cohort. They gave birth to 18329 babies, including 289 pairs of twins. Follow-up is ongoing. The main surveys so far were done by phone interviews when the child was aged 2 months, 1, 2, 3.5 and 5.5 years

Ethical approvals for each data collection wave during follow-up are obtained from the national advisory committee on information processing in health research (CCTIRS), the national data protection authority (CNIL) and, in case of invasive data collection such as biological sampling, the committee for protection of persons engaged in research (CPP). The Elfe study has also been approved by the national committee for statistical information (CNIS).

EDEN

EDEN (Etude des Déterminants pré et post natals du Dével-oppement et de la Santé de l'Enfant) is a French mother-child cohort study that, between 2003 and 2006, recruited 2002 pregnant women (<24 gestational weeks) aged 18–45 years at Nancy and Poitiers university hospitals. Exclusion criteria were twin pregnancy, history of diabetes, French illiteracy, and planning to move outside the delimited recruitment sites in the next three years.

Approval for the study was obtained from the Bicêtre Hospital ethics committee (Comité Consultatif de Protection des Personnes dans la Recherche Biomédicale: ID 02-20) and the National Committee for Processed Data and Freedom (Commission Nationale de l'Informatique et des Libertés: ID 902267). Written consent was obtained from each participant.

Supplementary Tables

Table S1. Piccolipiù prediction model (data: EUSILC-Italy 2011)

	**Coefficients**	**95% Confidence Interval**	
		R^2=0.44	
**Single mothers**	- 0.40	- 0.48	- 0.33
**Tenure status**			
owner	- -		
tenant	- 0.13	- 0.17	- 0.09
for free	- 0.14	- 0.19	- 0.10
**Number of rooms**			
1- 2	- -		
3	0.03	- 0.02	0.07
4	0.03	- 0.02	0.08
5	0.11	0.04	0.17
6+	0.09	0.02	0.17
**Household size**			
2	- -		
3	- 0.04	- 0.12	0.05
4	- 0.12	- 0.21	- 0.03
5	- 0.18	- 0.27	- 0.08
6+	- 0.24	- 0.35	- 0.12
**Maternal country**			
IT	- -		
EU	- 0.11	- 0.18	- 0.04
OTH	- 0.09	- 0.15	- 0.04
**Paternal/partner education**			
<=primary	- 0.20	- 0.29	- 0.10
lower secondary	- 0.07	- 0.11	- 0.04
upper secondary	- -		
>=post-secondary	0.12	0.07	0.18
**Maternal education**			
<=primary	- 0.16	- 0.25	- 0.07
lower secondary	- 0.05	- 0.09	- 0.02
upper secondary	- -		
>=post-secondary	0.06	0.02	0.10
**Paternal /partner occupation**			
employed	- -		
self-employed	- 0.09	- 0.13	- 0.04
unemployed	- 0.42	- 0.50	- 0.33
other	- 0.20	- 0.32	- 0.08
**Maternal occupation**			
employed	- -		
self-employed	- 0.02	- 0.07	0.03
unemployed	- 0.31	- 0.38	- 0.24
domestic tasks	- 0.43	- 0.48	- 0.38
other	- 0.22	- 0.34	- 0.11
**Paternal /partner ISCO88**			
0	0.01	- 0.05	0.07
1	- 0.01	- 0.08	0.07
2	0.07	0.00	0.13
3	0.01	- 0.05	0.07
4	- -		
5	- 0.10	- 0.18	- 0.02
6+7	- 0.06	- 0.11	0.00
8	0.02	- 0.04	0.07
9	- 0.16	- 0.23	- 0.09

**Maternal ISCO88**				
	1	- 0.08	- 0.16	0.00
	2	0.01	- 0.04	0.06
	3	0.01	- 0.04	0.06
	4	- -		
	5	- 0.10	- 0.16	- 0.04
	6+7+8	- 0.06	- 0.13	0.01
	9	- 0.22	- 0.29	- 0.14
**Paternal /partner age**		0.00	- 0.00	0.01
**Maternal age**		0.01	0.000	0.01
**Constant**		7.31	7.16	7.46

Table S2. NINFEA prediction model (data: EUSILC-Italy 2011)

	Coefficients	95% Confidence Interval	
		R^2=0.41	
**Single mothers**	- 0.37	- 0.44	- 0.30
**Dwelling type**			
Detached	- -		
Semi-detached	0.05	0.01	0.09
Flat	0.05	0.02	0.09
**Number of rooms**			
1-2	- -		
3	0.05	0.00	0.09
4	0.07	0.02	0.12
5+	0.15	0.09	0.21
**Household size**			
2	0.09	0.00	0.19
3	0.07	0.04	0.11
4	- -		
5	- 0.06	- 0.10	- 0.01
6+	- 0.12	- 0.20	- 0.04
**Maternal country**			
IT	- -		
EU	- 0.13	- 0.21	- 0.06
OTH	- 0.13	- 0.19	- 0.07
**Paternal/partner education**			
<=primary	- 0.23	- 0.32	- 0.14
lower secondary	- 0.11	- 0.14	- 0.07
upper secondary	- -		
>=post-secondary	0.15	0.10	0.20
**Maternal education**			
<=primary	- 0.18	- 0.28	- 0.08
lower secondary	- 0.06	- 0.09	- 0.02
upper secondary	- -		
>=post-secondary	0.07	0.03	0.11
**Paternal/partner occupation**			
Employed/self-employed	- -		
unemployed	- 0.36	- 0.44	- 0.28
other	- 0.13	- 0.24	- 0.01
**Maternal occupation**			
employed	- -		
self-employed	- 0.04	- 0.09	0.01
unemployed	- 0.33	- 0.40	- 0.26
domestic tasks	- 0.45	- 0.50	- 0.40
other	- 0.25	- 0.37	- 0.13
**Maternal ISCO88**			
1	- 0.07	- 0.15	0.01
2	0.02	- 0.04	0.07
3	0.01	- 0.04	0.06
4	- -		
5	- 0.12	- 0.18	- 0.05
6+7+8	- 0.05	- 0.12	0.02
9	- 0.24	- 0.32	- 0.17
**Maternal age**	0.01	0.01	0.01
**Constant**	7.01	6.89	7.12

Table S3. ELFE prediction model (data: EUSILC-Italy 2011)

	Coefficients	95% Confidence Interval	
		R^2=0.52	
**Single mothers**	- 0.31	- 0.38	- 0.24
**Separated/Divorced/Widow**	0.04	- 0.02	0.10
**Number of rooms**			
1- 2	- -		
3	0.09	0.00	0.18
4	0.14	0.05	0.22
5	0.15	0.06	0.24
6+	0.15	0.06	0.24
**Dwelling type**			
House	- -		
Flat in a big building	0.06	0.02	0.10
Flat in a small building	0.03	- 0.01	0.07
**Tenure status**			
owner	- -		
owner with mortgage	0.01	- 0.04	0.06
tenant	- 0.08	- 0.14	- 0.01
reduced rate	- 0.15	- 0.21	- 0.09
for free	- 0.02	- 0.11	0.08
**Household size**			
2	- -		
3	- 0.06	- 0.13	0.01
4	- 0.15	- 0.23	- 0.08
5	- 0.19	- 0.27	- 0.11
6+	- 0.21	- 0.31	- 0.12
**Paternal/partner country**			
FR	- -		
EU	- 0.02	- 0.11	0.07
OTH	- 0.08	- 0.14	- 0.02
**Maternal country**			
FR	- -		
EU	0.06	- 0.03	0.16
OTH	- 0.09	- 0.15	- 0.02
**Paternal /partner education**			
<=primary	- 0.05	- 0.11	0.01
lower secondary	0.01	- 0.04	0.06
upper secondary	- -		
>=post-secondary	0.09	0.05	0.12
**Maternal education**			
<=primary	- 0.07	- 0.13	- 0.01
lower secondary	- 0.07	- 0.11	- 0.03
upper secondary	- -		
>=post-secondary	0.10	0.06	0.13
**Paternal /partner occupation**			
employed	- -		
self-employed	- 0.12	- 0.19	- 0.06
unemployed	- 0.22	- 0.29	- 0.15
other	- 0.21	- 0.31	- 0.11
**Maternal occupation**			
employed	- -		
self-employed	- 0.03	- 0.11	0.05
unemployed	- 0.24	- 0.31	- 0.17
domestic tasks	- 0.27	- 0.33	- 0.22
other	- 0.26	- 0.31	- 0.20
**Maternal ISCO88**			

1	0.20	0.12	0.28
2	0.09	0.04	0.14
3	0.06	0.02	0.11
4	- -		
5	- 0.05	- 0.09	- 0.01
6+7+8	- 0.03	- 0.09	0.04
9	- 0.13	- 0.18	- 0.07
**Paternal /partner ISCO88**			
1	0.18	0.10	0.25
2	0.17	0.10	0.23
3	0.02	- 0.03	0.08
4	- -		
5	- 0.02	- 0.09	0.05
6	- 0.12	- 0.22	- 0.01
7	- 0.01	- 0.06	0.05
8	- 0.03	- 0.09	0.03
9	0.01	- 0.05	0.08
**Paternal /partner age**	0.00	0.00	0.00
**Maternal age**	0.00	0.00	0.01
**Constant**	7.22	7.06	7.38

Table S4. EDEN prediction model (data: EUSILC-Italy 2011)

	Coefficients	95% Confidence Interval	
		R^2=0.51	
**Single mothers**	- 0.30	- 0.37	- 0.23
**Separated/divorced/widow**	0.05	- 0.01	0.10
**Number of rooms**			
1-2	- -		
3	0.09	0.01	0.18
4	0.13	0.04	0.21
5	0.13	0.05	0.22
6+	0.13	0.04	0.22
**Tenure status**			
owner	- -		
tenant	- 0.11	- 0.14	- 0.08
for free	- 0.03	- 0.12	0.05
**Household size**			
2	- -		
3	- 0.07	- 0.14	0.01
4	- 0.16	- 0.24	- 0.09
5	- 0.20	- 0.28	- 0.12
6+	- 0.22	- 0.32	- 0.13
**Paternal/partner country**			
FR	- -		
EU	- 0.06	- 0.03	0.15
OTH	- 0.08	- 0.15	- 0.02
**Maternal country**			
FR	- -		
EU	0.06	- 0.03	0.15
OTH	- 0.08	- 0.15	- 0.02
**Paternal/partner education**			
<=primary	- 0.04	- 0.10	0.02
lower secondary	0.02	- 0.03	0.07
upper secondary	- -		
>=post-secondary	0.10	0.06	0.14
**Maternal education**			
<=primary	- 0.06	- 0.12	0.00
lower secondary	- 0.06	- 0.11	- 0.02
upper secondary	- -		
>=post-secondary	0.10	0.06	0.13
**Paternal/partner occupation**			
Employed/self-employed	- -		
unemployed	- 0.21	- 0.29	- 0.14
other	- 0.20	- 0.31	- 0.10
**Maternal occupation**			
Employed/self-employed	- -		
unemployed	- 0.25	- 0.31	- 0.18
domestic tasks	- 0.28	- 0.33	- 0.22
other	- 0.26	- 0.32	- 0.21
**Maternal ISCO88**			
1+2	0.12	0.07	0.17
3	0.06	0.02	0.11
4	- -		
5	- 0.05	- 0.10	- 0.01
6+7+8	- 0.05	- 0.11	0.02
9	- 0.13	- 0.18	- 0.07
**Paternal/partner ISCO88**			

1+2	0.15	0.09	0.22
3	0.02	- 0.04	0.07
4	- -		
5	- 0.02	- 0.09	0.05
6	- 0.19	- 0.29	- 0.09
7	- 0.03	- 0.09	0.02
8	- 0.03	- 0.09	0.02
9	0.02	- 0.05	0.08
**Paternal/partner age**	0.00	0.00	0.00
**Maternal age**	0.00	0.00	0.01
**Constant**	7.27	7.12	7.41

Supplementary Figures

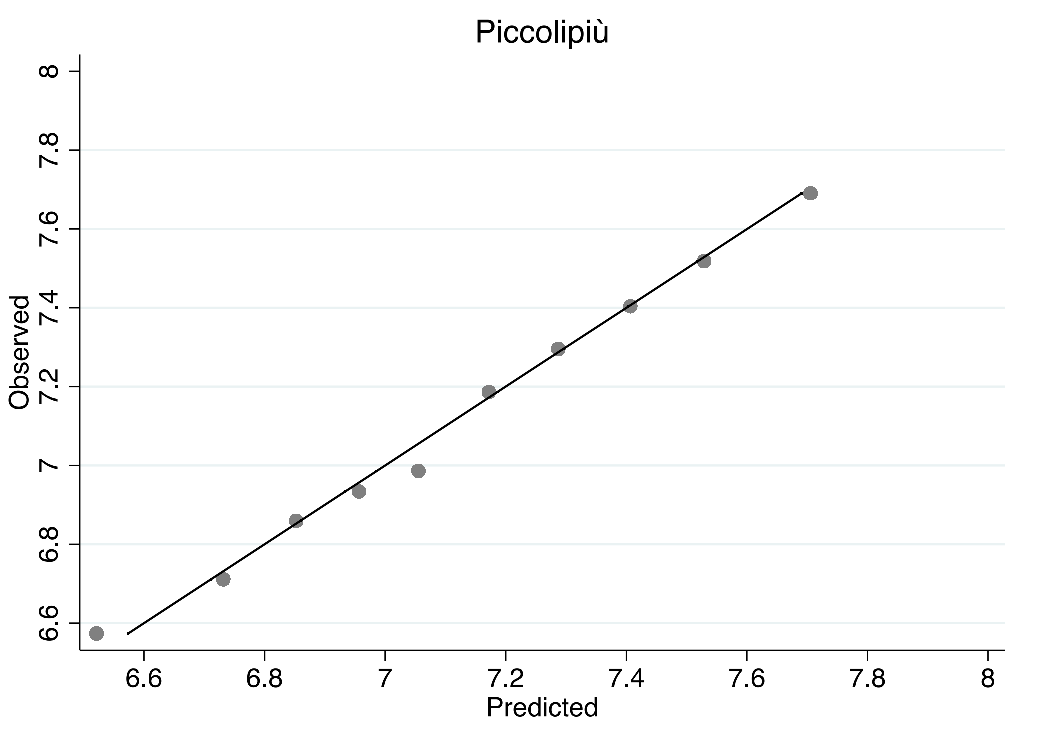

Fig. S1 Calibration plot for the Piccolipiù prediction model (log-income).

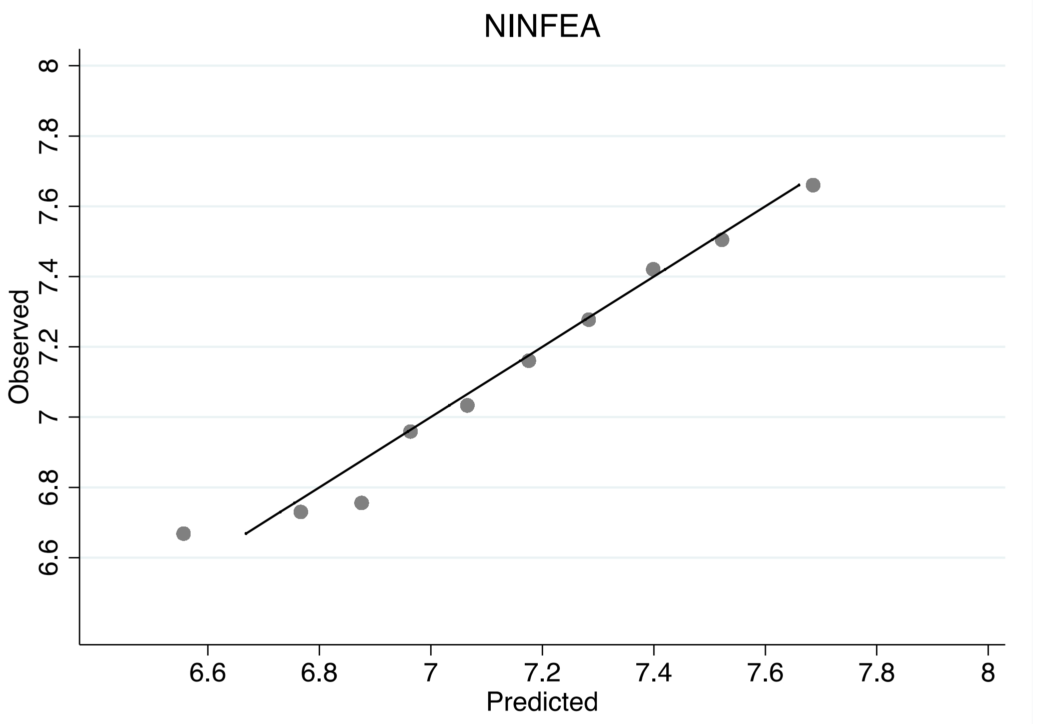

Fig. S2 Calibration plot for the NINFEA prediction model (log-income).

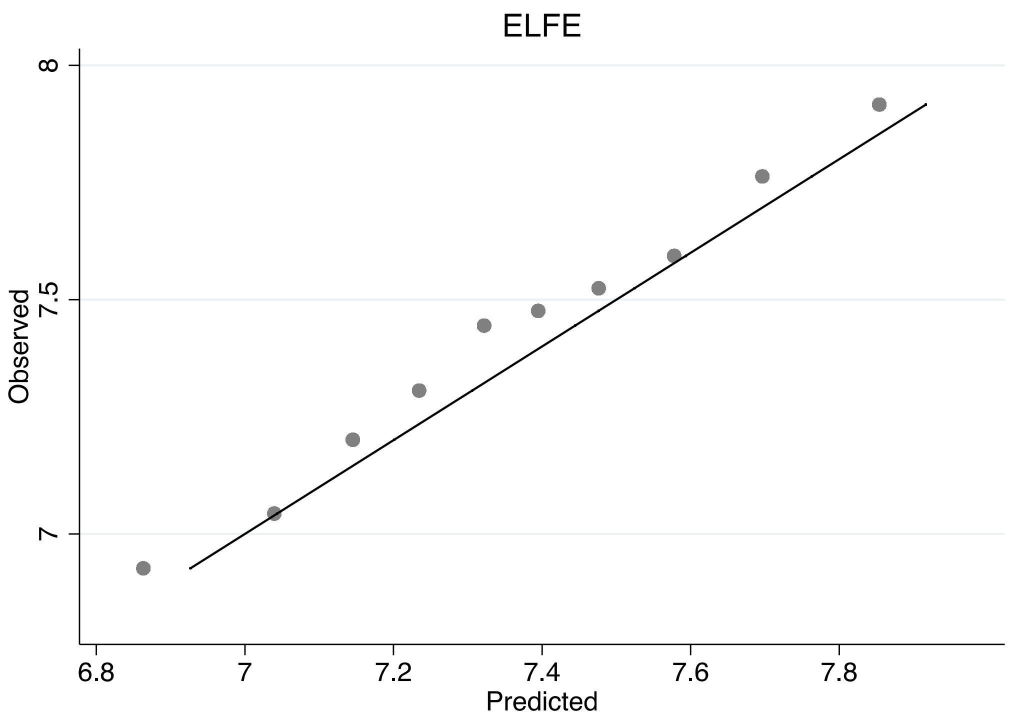

Fig. S3 Calibration plot for the ELFE prediction model (log-income).

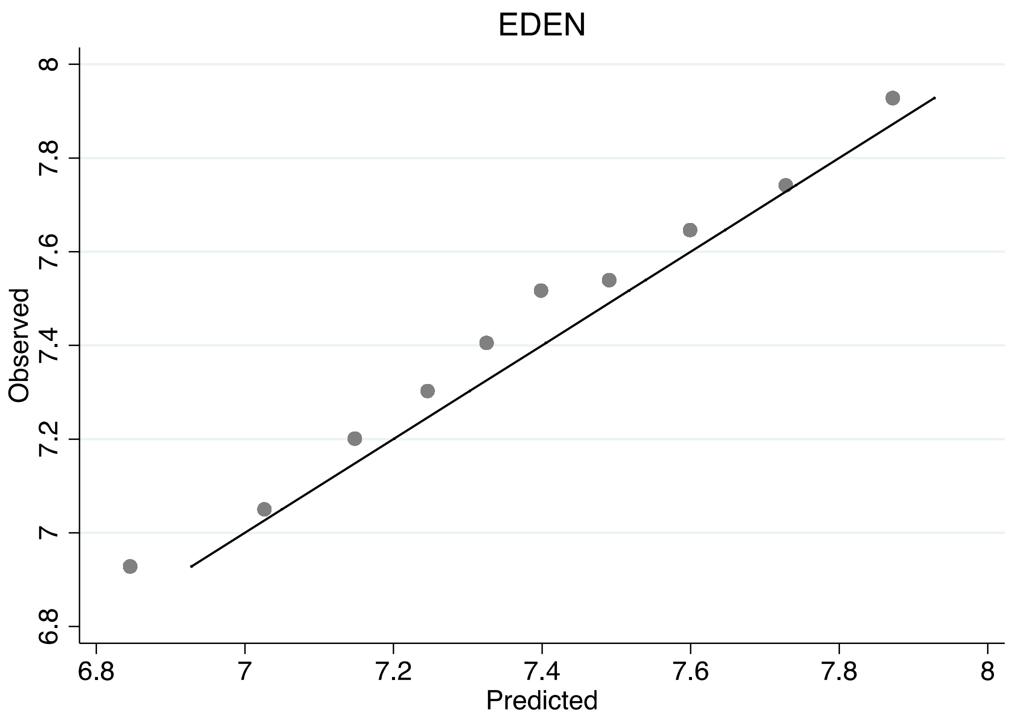

Fig. S4 Calibration plot for the EDEN prediction model (log-income).
